# Epidemiological study of drowning on coastal line of Bandar-e Anzali from 2015 to 2017 

**Published:** 2019-07

**Authors:** Hajar Khatami, Aref Abedin Najafi, Abdolreza Kazemzadeh, Mohammadreza Hadiani, Hajar Gholaminezhad, Ebrahim Asadi

**Affiliations:** ^*a*^MSc in Health Education, Alborz University of Medical Sciences, Alborz, Iran.; ^*b*^Head of Health Network, Bandar-e-Anzali, Guilan, Iran.; ^*c*^Disaster and Risk Management Specialist in Bandar-e-Anzali Health Network, Guilan, Iran.; ^*d*^Disease Specialist, Bandar-e-Anzali, Guilan, Iran.; ^*e*^Non-communicable Diseases Specialist, Deputy of Health, Guilan University of Medical Sciences, Rasht, Iran.; ^*f*^Head of Legal Medicine, Anzali, Guilan, Iran.

## Abstract

**Background:**

Drowning is one of the preventable unintentional events that causes many people all around the world to lose their lives every year. Bandar-e Anzali’s coastal line is one of the most popular areas in Iran whose dreadful drowning accidents made us take steps to prevent and reduce drowning burden through evaluating the most recent accidents in this realm.

**Methods:**

This research is an analytical-descriptive study which was conducted by analyzing 87 drowning cases that had happened in Bandar-e Anzali coast line during 3 years (2015- 2017). The data for this study were collected based on the drowning report forms in Anzali Health Network as well as the drowning death records of Anzali Legal Medicine, and were analyzed by means of SPSS 19.

**Results:**

In this study, the highest drowning rate was observed in 2015. Although there was a decrease in drowning in 2016, this rate changed to increase in 2017. 80.5% of drowning victims were men and 19.5% were women, and more than 55% of this led to death. 85.5% of the deceased were men and 75% of them were younger than 40 years old. More than 95% of drowning occurred outside the patrolled zone. Furthermore, more than 77% of drowning-related accidents occurred in summer. Tourists visiting Anzali accounted for more than 50% of the victims.

**Conclusions:**

Since the most number of accidents has occurred outside the patrolled zone, it is suggested to widen the coastal health zones, install warning signs out of the zones, and employ the skilled personnel to assist swimmers in the coastal lines.

**Keywords:**

Drowning, Bandar-e Anzali, Unintentional events

